# Targeting Viral Methyltransferases: An Approach to Antiviral Treatment for ssRNA Viruses

**DOI:** 10.3390/v14020379

**Published:** 2022-02-12

**Authors:** Peter Ramdhan, Chenglong Li

**Affiliations:** Department of Medicinal Chemistry, College of Pharmacy, University of Florida, Gainesville, FL 32603, USA; lic@cop.ufl.edu

**Keywords:** methyltransferase inhibitors, methyltransferase, KDKE tetrad

## Abstract

Methyltransferase enzymes have been associated with different processes within cells and viruses. Specifically, within viruses, methyltransferases are used to form the 5′cap-0 structure for optimal evasion of the host innate immune system. In this paper, we seek to discuss the various methyltransferases that exist within single-stranded RNA (ssRNA) viruses along with their respective inhibitors. Additionally, the importance of motifs such as the KDKE tetrad and glycine-rich motif in the catalytic activity of methyltransferases is discussed.

## 1. Introduction

The denotation of a protein as a methyltransferase is a general term for the enzymatic activity of a protein transferring a one-carbon group onto a select substrate. These substrates and the role that they play can vary based on the organism and the viral genome that codes for that particular methyltransferase. This review seeks to categorize a majority of the known methyltransferases within the viral world to explore their role and implications in drug design.

## 2. Background Information

Viruses can be further split into categories based on the genome that they possess [1]. From there, the proteins that are coded tend to be somewhat similar amongst the different subfamilies.

### 2.1. Baltimore Classification System

Viruses can be classified using the Baltimore classification system [1]. This classification system is based on their genetic material which subsequently affects their life cycle. In Table 1 below, displays the families of viruses in broad categories accompanied by common examples.

### 2.2. General Life Cycle

**Figure 1:**
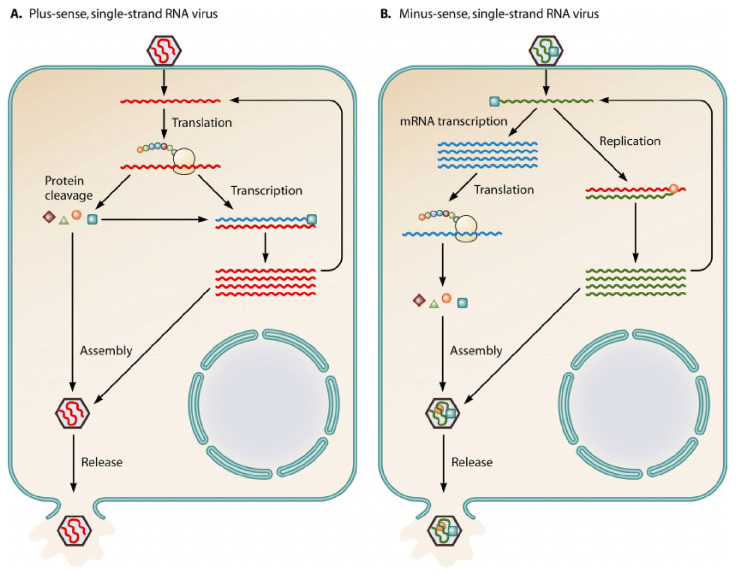
The above diagram displays the notable differences between a (+)—sense single-stranded RNA virus and a (−)—sense single-stranded RNA virus [2].

Methyltransferases are present within both Class 4 and Class 5 viruses [2]. Once Class 4 and Class 5 viruses gain entry intro the cell, they utilize different methods of replication which is portrayed in Figure 1 below.

The following are positive-sense single-stranded RNA viruses that are further reviewed in this paper: *SARS-CoV, Berne virus, Zika virus, Rubella virus, Semliki virus,* and *Hepatitis E.* In this method of replication, the virus enters the cell and releases its genomic material. Since the positive-sense strand is of the same nature as what mRNA would be, the ribosome of the host cell would be able to effortlessly translate the viral proteins to continue its life cycle. It can thus create its polymerase, coupled with cleavage of its genome, to develop additional proteins needed for its life cycle. Once maturation is achieved, the virus is packaged and released for infection of another cell.

The following are negative-sense single-stranded RNA viruses: *Nishimuro ledantevirus, rabies virus,* and *Human Respiratory Syncytial virus A*. In this type of replication, the virus releases its contents into the cell. Since this strand is not of the same nature as mRNA, the virus codes for the RNA-dependent RNA polymerase to convert the negative-sense RNA to a positive-sense mRNA for translation. The virus then follows similar subsequent steps as the positive-sense single-stranded RNA virus. This cycle begins anew with the release of the newly packaged viral material.

### 2.3. Methyltransferases: Cap Formation

Methyltransferases are mainly seen to be utilized within cap formation [3]. Generally, cap formation will involve the following processes: an RNA-triphosphatase (TPase), RNA-guanylyltransferase (GTase), then guanine-N7-methyltransferase. The activation of a G-N7-methyltransferase creates the Cap-0 structure [4]. The purpose of these cap structures is to give the host cell the impression that the invading viral genome is of host origin. By disguising this mRNA strand, the virus can evade the host’s innate immune system and go on to further replicate and invade more cells.

### 2.4. Methyltransferases: Restriction-Modification System

In the realm of *bacteria*, restriction-modification systems are used to rid the cell of foreign DNA [5]. The system is composed of a restriction endonuclease (REase) and a methyltransferase (MTase). The REase is responsible for recognizing short specific DNA sequences or recognition sites and then cleaves them. The methyltransferase is responsible for methylating certain sequences which protects specific areas of the DNA from endonucleolytic cleavage via REase.

The restriction-modification system can be divided into four main types: Type 1, Type 2, Type 3, and Type 4. These are categorized according to subunit composition, cofactor requirements, and other properties.

### 2.5. Methyltransferase Categories

Methyltransferases are further split into multiple categories based on their structural features, such as the catalytic domain and structural motifs that comprise them. While not all methyltransferases can be confined to a single category, a majority subside under the larger umbrella of Class 1 methyltransferases. A generalized classification of methyltransferases and their corresponding names can be found in Table 2 below. Methyltransferases can either utilize S-adenosyl-L-methionine (SAM) or a different cofactor, such as THF, the latter being less common amongst biological mechanisms [6].

## 3. Methyltransferase Discussion

The following list encompasses methyltransferases that have their substrate as single-stranded RNA. Therefore, only methyltransferases involving Class 4, 5, and 6 of the Baltimore classification system will be discussed. In Table 3, viral representatives are chosen for both positive-sense and negative-sense ssRNA viruses along with their respective uniport reference ID. These viral representatives are expounded upon further within this review. In Table 4, the viral methyltransferases are further discussed along with statement of their reaction substrates, purpose of the methyltransferase in the viral life cycle, and the methyltransferase class that they belong to.

### 3.1. Genome Sequencing and Similarity by Domains

**Figure 2:**
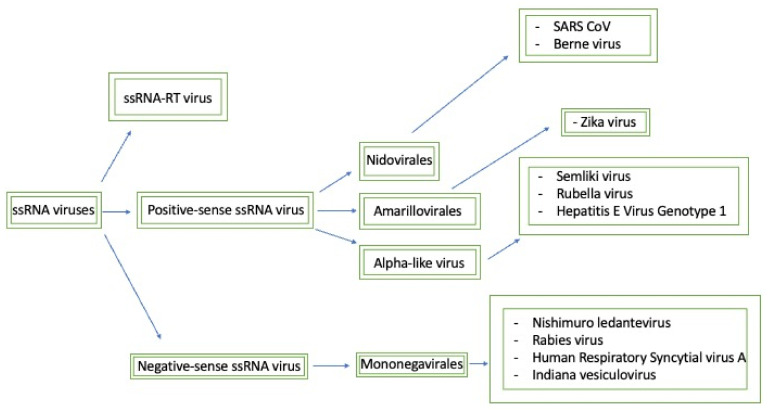
This map seeks to briefly summarize the methyltransferases that will be discussed and how they relate to each other. Generally, the negative-sense ssRNA viruses have similar methyltransferase domains that are classified as mononegavirales-like methyltransferases. Furthermore, based on the sequence domain composition, positive-sense ssRNA viruses can be split into three separate families: nidovirales-like, NS5-like, and alpha-like methyltransferases.

Figure 2 connects the methyltransferase domains amongst their respective ssRNA classes and displays both the domain sequences and motifs that are essential for activity.

### 3.2. PDB Structures with the Active Site

**Figure 3:**
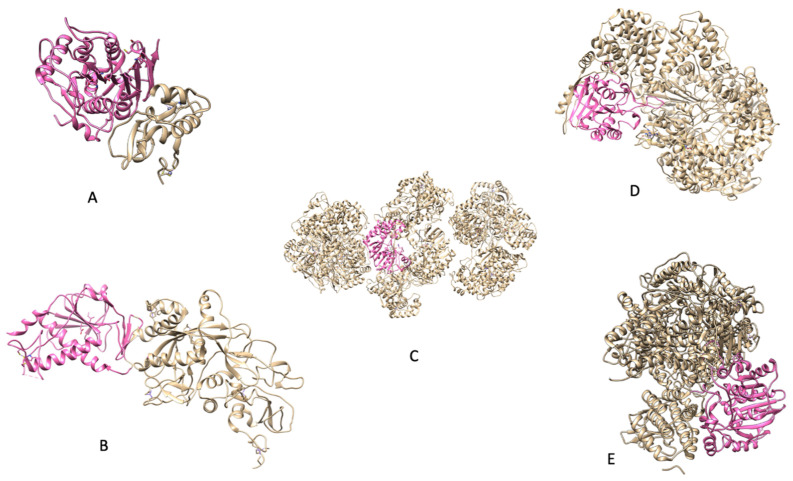
(**A**) PDB 3R24: NSP16 protein from *SARS CoV*); (**B**) (PDB 5C8U: NSP14 protein from *SARS CoV*); (**C**) (PDB 5M2X: NS5 protein from *Zika*); (**D**) [8] (PDB 5A22: Protein L from *Indiana vesiculovirus*); (**E**) (PDB 6UEB: L protein from *Rabies virus*). *Indiana vesiculovirus* was used as its sequence is quite similar to that of other negative-strand ssRNA viruses and PDBs are not available for the viruses discussed.

In Figure 3, there are key examples of methyltransferases that are well-known along with their methyltransferase domains.

Not all viruses have one protein dedicated solely to acting as a methyltransferase. Some methyltransferases have to make do with what they have and form bifunctional proteins such as that of *SARS-CoV’s* NSP14. This makes the replication and infection process much simpler for the virus if their genome is not large. The above image displays the available PDB files for the methyltransferases that have been discussed so far. The bright pink highlight seeks to partition the methyltransferase domain from the rest of the protein. While the methyltransferase domain is highlighted above, the domain can be further explored as there are specific motifs that are conserved amongst these proteins.

### 3.3. KDKE Motif

**Figure 4:**
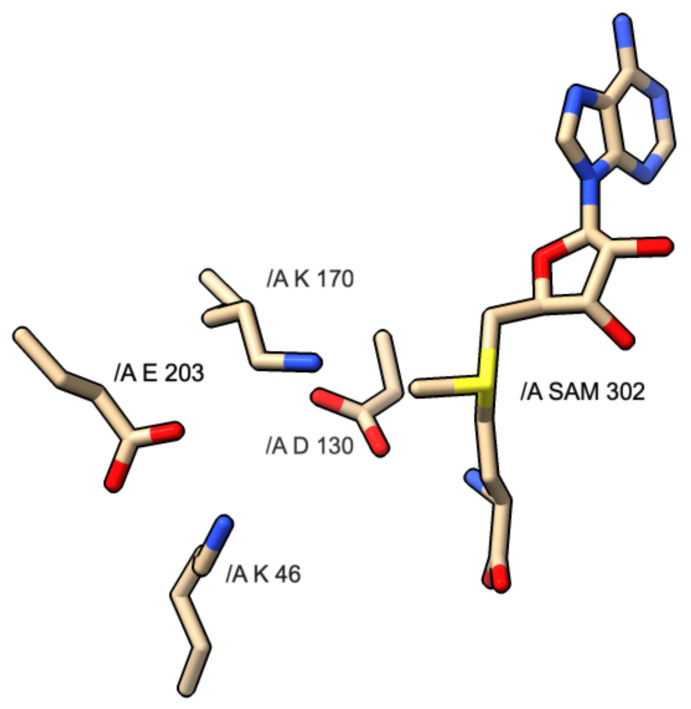
Diagram of KDKE motif and interaction with SAM molecule found in PDB 3R24: *SARS CoV-1* NSP16 protein.

The KDKE motif is comprised of four residues involved with the catalytic exchange of a methyl group onto the cap-acceptor site of the RNA strand. Figure 4 shows the KDKE tetrad interacting with the SAM molecule. The yellow shaded portions represent sulfur atoms, the blue shaded portions represent nitrogen atoms and the red shaded atoms represent oxygen atoms.

**Figure 5:**
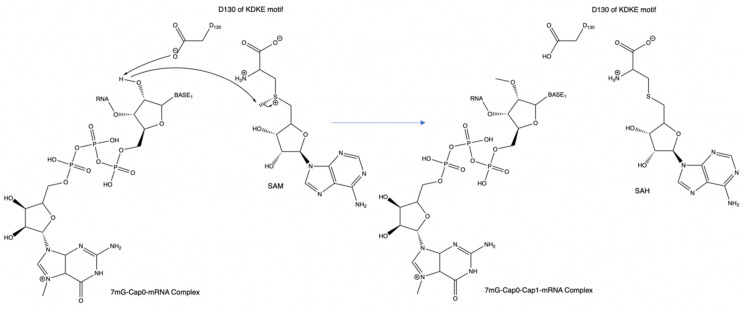
An example of the mechanism of the KDKE motif within PDB 3R24. This can also be considered the general mechanism for methyl transfer onto a substrate.

This motif is more common with the formation of cap-1 instead of cap-0, as most proteins responsible for sole cap-0 formation lack this motif. The residues involved are two lysines, one aspartic acid, and one glutamic acid. While this motif is very common, some variations can exist that are capable of catalyzing the same mechanism. In the above images, the KDKE motifs are displayed within their respective PDB files. In PDB 5M2X, the KDKE motif is K61, D146, K182, and E218. In PDB 3R24, the KDKE motif is K46, D130, K170, and E203. In PDB 6UEB, the KDKE motif is K1685, D1797, K1829, E1867. In PDB 5A22, the KDKE motif is K1651, D1762, K1795, and E1833. Figure 5 shows an example of the mechanism of methyl transfer from SAM to RNA.

The aspartic acid (D) within the KDKE motif is responsible for the initiation of the cap structure formation. The two lysine residues have a possible role in stabilizing the incoming RNA molecule as it prepares to be methylated. This interaction involves the positively charged lysine residues and the negatively charged phosphate backbone of the RNA strand. Regarding the possible role of the glutamic acid residue, its role could be associated with maintaining the structural integrity of the RNA binding pocket by retaining the two lysine residues in a position where it can optimally interact with the RNA phosphate backbone. In Figure 5, PDB 3R24 displays an RNA molecule with a Cap-0 structure or methylated N7 atom on the guanine nucleotide. PDB 3R24 shows NSP16 of SARS-CoV which is responsible for utilizing Cap-0 and SAM to form the Cap-1 structure. D130 will act as a Lewis base to deprotonate the ribose-2′OH. During this deprotonation, the ribose-2′O anion will simultaneously attack the methyl group via an SN2 attack. This reaction converts the S-adenosyl-L-methionine to S-adenosyl-homocysteine.

In the case of *SARS-CoV* and NSP16, NSP16 has a strong preference for the Cap0-mRNA complex as a substrate rather than an mRNA substrate without the Cap0 structure. To understand the selectivity of this cap-dependent process, one must first compare the difference between normal guanine and the 7-methyl guanine. While the guanine is composed of a bicyclic delocalized ring, the 7-methyl guanine has the same components with the added benefit of a methyl which grants the 7mG group a positive charge. The type of bond between this positive charge and the aromatic residue seen in a majority of these RNA-binding domains can be described as a cation–pi interaction. The most common pi system involved in this type of interaction is typically tryptophan and is seen to be energetically favorable. Based on the characterization of the interaction of methylated N7 vs. non-methylated N7 within the EIF4E binding protein by the Brown lab, it was concluded that methylated N7 was calculated to have a tighter binding to the cap-0 structure vs. no cap at all [9]. This explains why some methyltransferases prefer cap structures present before additional methylation and subsequent cap formation.

### 3.4. Glycine-Rich Sequence Motif

**Figure 6:**
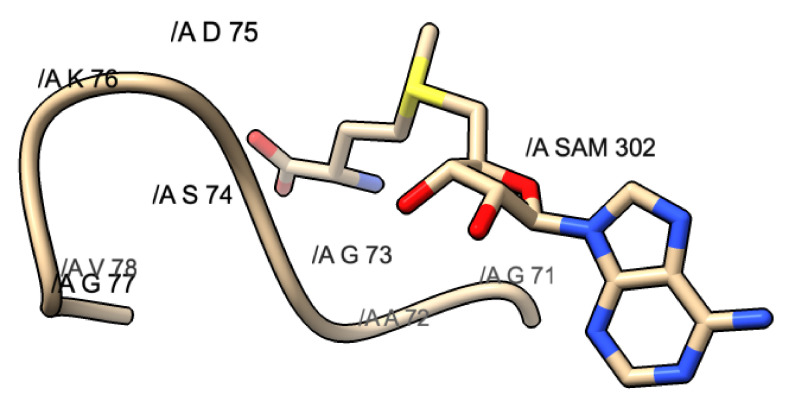
GxG motif found in PDB 3R24: SARS-CoV. This motif is composed of G71, A72, and G73. The yellow shaded portion represents the methionine portion of SAM, the blue represents the nitrogen, and the red represents the oxygen atoms.

Another common motif that is found within methyltransferase domains is the glycine-rich motif. This can come in the form of DxG, GxG, GxGxG, or some other variation. This motif usually will mark the area where SAM will dock within the methyltransferase domain. Figure 6 shows an example of the GxG motif found underneath the methionine portion of the SAM molecule.

The methionine portion of the molecule will dock above this motif and stabilize via small hydrophobic interactions seen between the SAM molecule and the motif. The purpose of this conservation most likely serves as a designated docking location for SAM.

**Figure 7:**
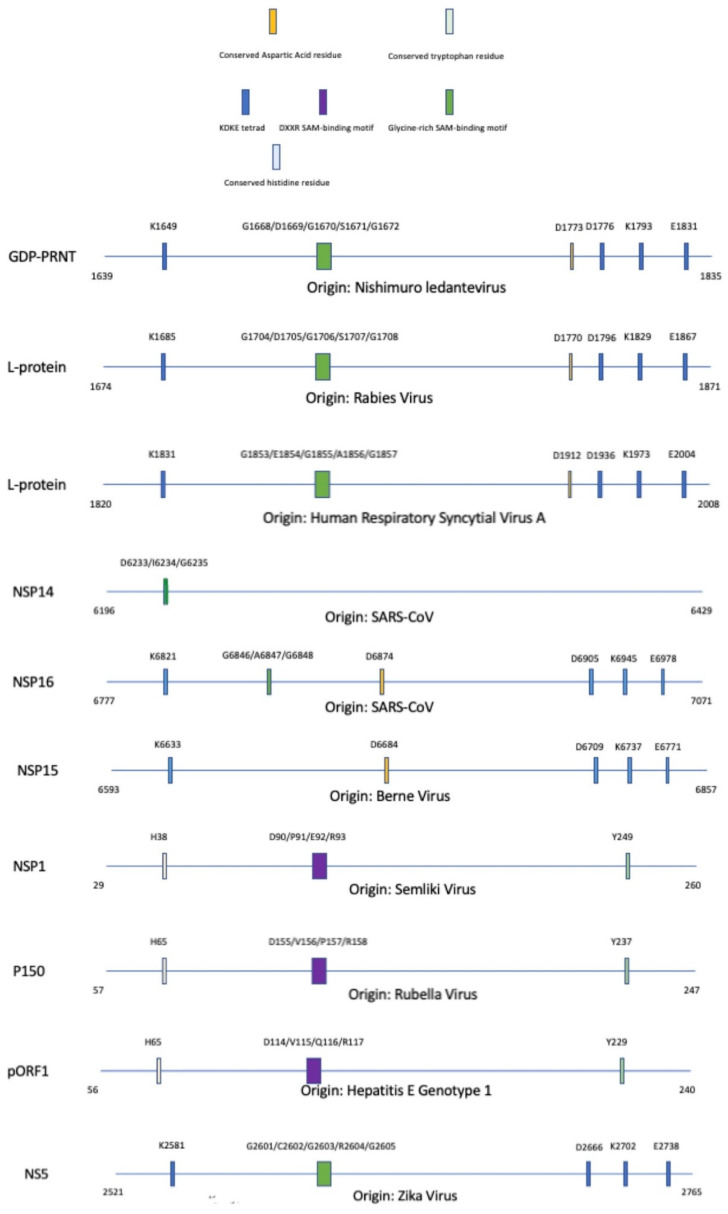
Alignment of methyltransferase domains and their unique motifs involved with the completion of enzymatic activity; SAM: S-adenosyl-L-methionine. A legend is provided to partition each methyltransferase based on specific motifs that are conserved within the protein sequence. The significance of these motifs are explained throughout this paper.

With an introduction to the main motifs seen within methyltransferases, the different domains can now be presented via the different classes that they are categorized in. The composite diagram below, Figure 7, separates the methyltransferase domains into subgroups via the proposed methyltransferase domain similarities as mentioned in Figure 2.

For the amarillovirales-like methyltransferase domain family, there appear to be conserved motifs such as the KDKE tetrad and the glycine-rich SAM-binding domain. For the alpha-like virus methyltransferase domain family, there appear to be conserved motifs such as a conserved histidine residue, DXXR SAM-binding motif, and conserved tryptophan residue. While the full mechanism is unclear, the conserved histidine and tryptophan residues are essential for methyltransferase activity and are shown to be associated with catalytic activity.

For the nidovirales-like methyltransferase domain family, we can see a conserved aspartic acid residue within NSP16 of *SARS-CoV* and NSP15 of *Berne,* but not in NSP14 of *SARS-CoV*. The KDKE tetrad is found within the NSP16 and NSP15, but not NSP14. A glycine-rich SAM-binding motif can be found conserved in NSP16 and NSP15. For the mononegavirales-like methyltransferase domain family, there appear to be conserved motifs such as KDKE tetrad, conserved aspartic acid residue, and glycine-rich SAM-binding motif. The conserved aspartic acid may play a role in interacting with the hydroxyl groups on the ribose of SAM to aid in retaining the molecule during methyl transfer. A more in-depth explanation of the motif composition is given in the virus’ respective subsections below.

## 4. Methyltransferase Domains and Their Representatives

### 4.1. Amarillovirales Methyltransferase Domain Family

#### NS5 (A0A024B7W1) *Zika*

**Figure 8:**
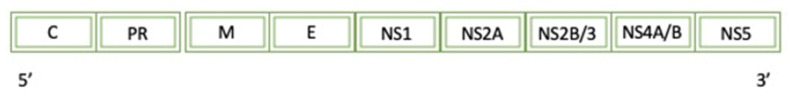
A diagram of the simplified genome for the *Zika Virus*: C (Capsid), PR (peptide PR), M (small envelope protein), E (envelope protein), and NS protein (non-structural protein).

*Zika* is a positive-sense ssRNA that enters the host cell via receptor-mediated endocytosis [10]. Figure 8 displays a simplified diagram of the full *Zika* genome. After translation of the viral proteins, NS5 plays an essential role in the life cycle of Zika. NS5 is composed of two domains: an N-methyltransferase domain and an RNA-dependent RNA polymerase. In this reaction, SAM is used as a cofactor to catalyze the transfer of a methyl group to its final acceptor. In vitro studies have shown that while the MTase domain is associated with transferring a methyl group to the N7 of guanine, the domain has been seen to transfer a methyl group to the ribose-2′OH and act as a guanylyltransferase.

Based on different mutagenesis studies, the following sites can be seen as important for functionality: mRNA cap-binding sites, SAM binding sites, and general sites that are seen to be essential for methyltransferase activity [11]. For mRNA cap-binding, the following residues are deemed important for activity as they are involved directly with binding: K2533, L2536, N2537, M2539, F2544, K2548, S2670, Y2733, and E2735. For SAM binding, the following residues are deemed important for activity as they are involved directly with binding: S2576, K2606, T2624, K2625, H2630, E2631, D2651, V2652, I2667, and Y2740.

For residues that are classified as being generally involved in methyltransferase activity, the following have been seen to exhibit this: K2581, D2666, K2702, and E2738. Additionally, a glycine-rich motif can be seen which can indicate the canonical SAM-binding domain. This can be defined as G2601/C2602/G2603/R2604/G2605.

### 4.2. Alphavirus-like Methyltransferase Domain Family

#### 4.2.1. NSP1 (P08411) *Semliki Virus*

**Figure 9:**
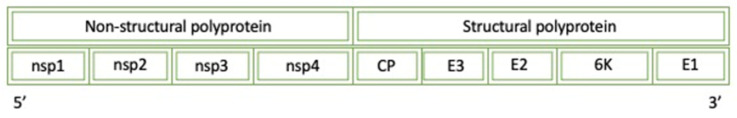
A diagram of the simplified genome for the *Semliki Virus*: CP (Capsid protease), E (envelope protein), NS (non-structural protein), and 6 K protein (6 kDa protein).

Semliki virus is a positive-sense ssRNA virus that undergoes early translation once in the cell [12]. Figure 9 displays a simplified diagram of the full *Semliki* genome. Proteins are synthesized to create a complex that is capable of cap formation. The cap will form solely in the nucleus; however, this virus replicates exclusively in the cytoplasm. RNA TPase will remove the 5′ gamma-phosphate of the nascent RNA molecule. mRNA guanylyltransferase will donate a GMP to form a 5′-5′ TP linkage. NSP1 will bind to GMP to form an enzyme-GMP complex. The GMP is transferred to mRNA where SAM is used to transfer a methyl group to guanine-N7 to form the cap structure.

There have been residues reported that are important in this enzyme’s catalytic activity. Residue 38H is involved in creating the initial phosphoramide link between the enzyme and the mRNA structure. The following residues are seen to interact with the adenine base of the mRNA strand: A39, D153, Y155, Y249, and Y286. The following residues are seen to interact with the triphosphate portion of the mRNA strand: R42, S45, H46, K50, T70, Q152, and D153. The following residues are seen to interact with SAM within the binding pocket: G66, R71, R72, P84, E89, D90, R93, Q152, and D153.

Additional site-directed mutagenesis studies further show how mutations in specific residues are needed for functionality. Specifically, within the methyltransferase domain, mutations in the following sites have shown decreased activity: L19, H38, D64, C81, V82, C83, D90, R93, C135, C142, K169, C214, Y249, and K317.

The methyltransferase domain of the *Semliki* virus is expected to have some key motifs that can be found within its sequence: an invariant histidine residue, a DXXR motif, and an invariant tyrosine residue. The histidine residue is found as H38, the DXXR motif as D90/P91/E92/R93, and the tyrosine residue is Y249.

#### 4.2.2. pORF1 (Q81862) *Hep E Virus Genotype 1*

**Figure 10:**
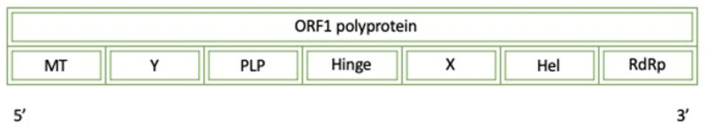
A diagram of the simplified genome for the ORF1 polyprotein of *Hepatitis E Virus*: MT (methyltransferase), Y (Y-domain), PLP (papain-like protease), X (X-domain), Hel (Helicase), and RdRp (RNA-dependent RNA polymerase).

The *Hepatitis E virus* is located within the family Hepeviridae and genus Orthohepevirus [12]. Figure 10 displays a simplified diagram of the full *Hepatitis E Virus Genotype 1* genome. This is a single-stranded positive-sense ssRNA that is transmitted via the fecal–oral route. Once viral particles are ingested, the virus is absorbed via GI mucosa and into portal circulation to enter the liver. The following regions are present in ORF1 polyprotein: methyltransferase (60–240), Y-domain (239–439), putative papain-like cysteine protease (440–610), hinge (712–778), X-domain (785–942), NTPase/helicase (960–1204), RdRP (1207–1693) [8].

The methyltransferase domain is expected to contain the following: an invariant histidine residue, a DXXR motif, and an invariant tyrosine residue [7]. The invariant histidine residue can be noted as H65, the DXXR motif as D114/V115/Q116/R117, and the invariant tyrosine residue as Y229. The DXXR motif is defined as Motif II and proposed to comprise a portion of the SAM-binding site [13,14]. An interesting characteristic that the genome possesses is the m7G cap and polyadenylated tail that is already present before the subsequent translation of the mRNA. This virus is thus protected from the host cell to ensure optimal translation.

#### 4.2.3. P150 (Q86500) *Rubella*

**Figure 11:**
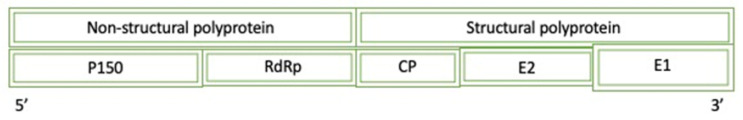
A diagram of the simplified genome for the *Rubella Virus*: P150 (protease/methyltransferase 150), RdRp (RNA-dependent RNA polymerase), CP (cysteine protease), and E (envelope).

*Rubella* is a positive-sense ssRNA virus where the virus initially binds to an unknown receptor of susceptible cells to catalyze receptor-mediated endocytosis and subsequent release of the genomic material into the cytoplasm of the host cell [1]. Figure 11 displays a simplified diagram of the full *Rubella* genome. The genomic RNA is translated to produce the non-structural polyprotein which is comprised of gene products after further cleavage, P150 and P90 (RdRP). P150 has three subdomains that are capable of: methyltransferase functionality, a domain of an unknown function, and proteolytic function. While the specifics of the methyltransferase are not further discussed, the function can be assumed to be related to cap formation as this domain is found in the polymerase of the virus.

Similar to that of the *Hepatitis E genome*, *Rubella* will also contain some key motifs within the methyltransferase domain such as an invariant histidine residue, a DXXR motif, and an invariant tyrosine residue. An invariant histidine residue can be found as H65, the DXXR motif as D155/V156/P157/R158, and an invariant tyrosine residue at Y237.

#### 4.2.4. Alignment of the Alpha-like Virus Methyltransferase Sequences

**Figure 12:**
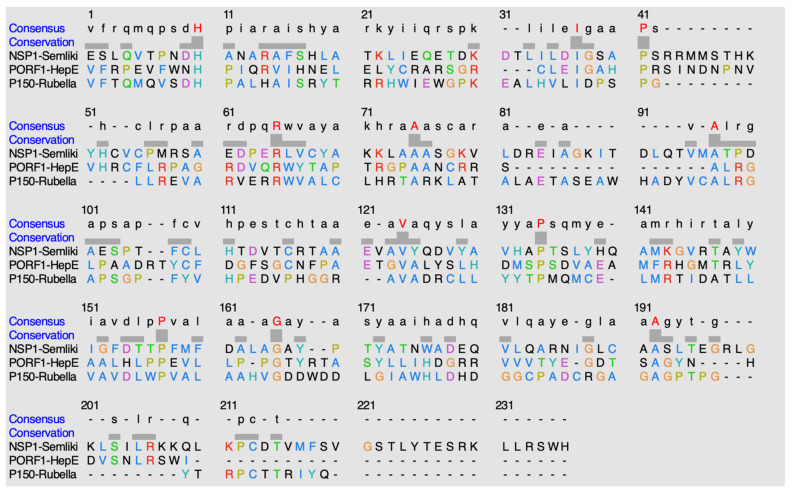
Alignment of sequences using ClustalO. The highlighted red residues in the consensus row display residues that are highly conserved amongst the methyltransferases. The hyphens indicate either no consensus residue or no residue present at all.

ClustalO is a sequence alignment software that is utilized to discover potential conservation of residues amongst the methyltransferase domains within this classification of methyltransferases, alpha-like methyltransferase domain [15]. Figure 12 shows the sequence alignment of the methyltransferases within this alpha-like methyltransferase family.

The following is found to be conserved within the methyltransferase domain of NSP1 in the *Semliki Forest Virus*: H38, I65, P69, R93, A103, A125, V150, P160, P183, G191, and A216. The following is found to be conserved within the methyltransferase domain of PORF1 in the *Hepatitis E virus*: H65, I89, P93, R117, A127, A134, V161, P171, P194, G201, and A227. The following is found to be conserved within the methyltransferase domain of P150 in the *Rubella virus*: H66, I93, P97, R109, A119, A141, V164, P174, P196, G204, and A231.

When comparing percent identity amongst the sequences, the values observed are less than 20%. When NSP1 (*Semliki*) is compared to PORF1 (*Hepatitis E*), 15.68% identity is observed. When NSP1 (*Semliki*) is compared to P150 (*Rubella*), 18.85% identity is observed. When PORF1 (*Hepatitis E*) is compared to P150 (*Rubella*), 17.84% identity is observed.

### 4.3. Nidovirus-like Methyltransferase Domain Family

#### 4.3.1. NSP14 and NSP16 (P0C6X7) *SARS-CoV*

**Figure 13:**
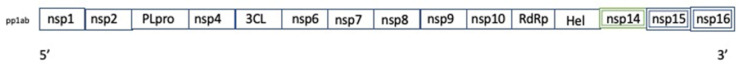
A diagram of the simplified genome for the *SARS-CoV Virus*: NS (non-structural protein), PLpro (papain-like protease), 3CL (3CL-like protease), and RdRp (RNA-dependent RNA polymerase), Hel (Helicase).

*SARS-CoV* is a positive-sense ssRNA virus that codes for a non-structural protein, NSP14, that has bifunctionality to act as both a 3′-to-5′ exoribonuclease and guanine-N7 methyltransferase [16]. Figure 13 displays a simplified diagram of the full *SARS-CoV* genome. *SARS* can code for an additional non-structural protein, nsp10, which only acts to enhance the activity of nsp14′s exoribonucleolytic function but not its methyltransferase activity. Based on studies cited in Reference 15, it is shown that dGTP can be recognized as a substrate but not dATP, dCTP, or dTTP. Additionally, dinucleotides such as GpppG, GpppA, or m7-GpppG can be recognized and methylated by the N7-Mtase domain. While the initial two are methylated, GpppG and GpppA, the methylated dinucleotide will not be methylated further. This further proves that NSP14 is specific for the N7 position of the first G nucleotide. NSP14 does not methylate G nucleotides internally within the RNA strand but only the G nucleotide at the front of the strand.

Based on the PDB 5C8U, the following residues are said to be extremely essential for the activity of the N7 MTase: F73, R84, W86, R310, D331, G333, P335, Y368, C414, C416 [17].

NSP16 acts as a ribose-2′OH methyltransferase that aids in the formation of cap-1 [18]. Similar to NSP14, NSP16 requires interaction with NSP10 to achieve maximum activity for its methyltransferase capabilities.

Common motifs seen with nidovirales-like methyltransferase domains are the glycine-rich motif or some variation, and KDKE tetrad, except NSP14. As mentioned before, KDKE tetrads are mainly associated with forming the cap-1 structure and not the cap-0 structure. The KDKE tetrad is thus not conserved in the NSP14 methyltransferase domain, but still viable in NSP16 and NSP15 of the other nidovirales-like methyltransferase domains mentioned in this section.

With discussing NSP14, the reported SAM-binding region is comprised of residues 6233–6239 [DIGNPKA] while the reported GpppA-mRNA strand interacts with the following residues: 6316–6330 [DGGSLYVNKHAFHT]. While this DxG (D6233/I6234/G6235) motif is not similar to the GxG motif, it is a slight variation of the SAM-binding motif.

With discussing NSP16, the following motifs are seen: GxG motif and KDKE tetrad. The GxG motif can be found at G6846/A6847/G6848 while the KDKE tetrad is found at K6821/D6905/K6945/E6978. When viewing PDB 3R24, a key SAM-binding residue (D6874), is essential for stabilizing and forming a sturdy hydrogen bond with the ribose hydroxyl groups to anchor the SAM molecule. Upon further inspection, this residue is conserved within close relative NSP15 from the *Berne virus*. This SAM-ribose binding residue can be defined as D6684. While this same residue seems to be conserved within NSP14, the residue is outside of the N7-methyltransferase domain and no further experimental data is available to confirm its participation in SAM-binding. It can be hypothesized that since the substrates of NSP14 are different from that of NSP14 and NSP15, this conserved aspartic acid residue may not play a role within the functionality of NSP14.

#### 4.3.2. NSP15 (P0C6V7) *Berne Virus (Equine Torovirus)*

**Figure 14:**
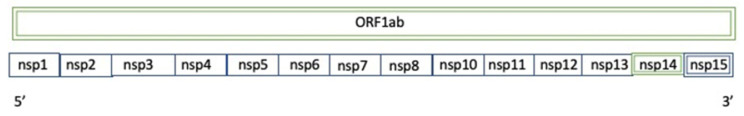
A diagram of the simplified genome of ORF1ab for the *Berne Virus*: NS (non-structural protein). NSP refer to the non-structural proteins. ORF refers to the open-reading frame.

The torovirus is a positive-sense ssRNA virus with a peplomer-bearing envelope.^17^ Since toroviruses are ancestrally similar to coronaviruses, there is some similar nomenclature amongst the gene names. Figure 14 displays a simplified diagram of the full *Berne* genome. The following genes are translated: ORF1ab is known as the replicase polyprotein, spike (S), membrane (M), hemagglutinin-esterase (HE), and nucleocapsid (N). The replicase polyprotein is cleaved into 14 chains: NSP 1–8, 10–15. NSP15 assumes the role of a methyltransferase. The domain is similar to that of other nidoviral-type SAM-dependent methyltransferases. This specifically will methylate the ribose-2′OH structure to form the cap-1 structure.

With discussing NSP15, the following motifs are seen: KDKE tetrad. The KDKE tetrad is found at K6633/D6709/K6737/E6771.

#### 4.3.3. Alignment of the Nidovirales-like Methyltransferase Sequences

**Figure 15:**
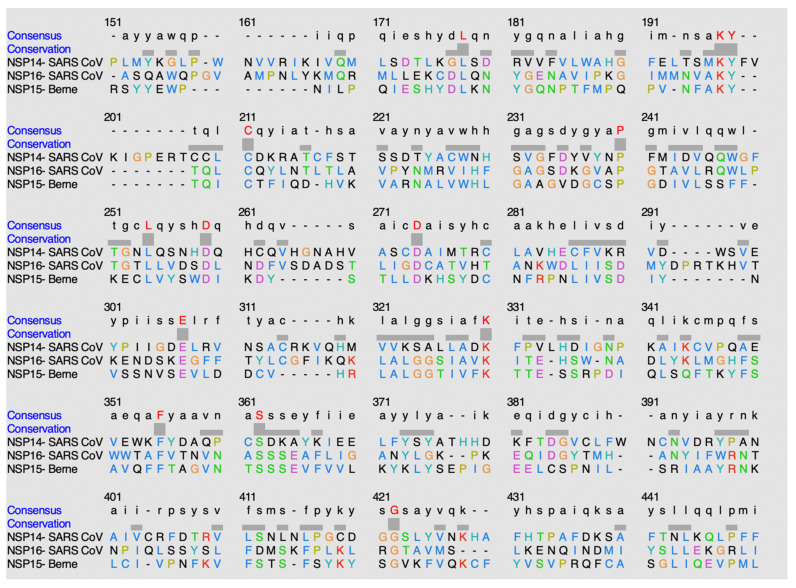
Alignment of sequences using ClustalO.

ClustalO is a sequence-alignment software that was utilized to discover potential conservation of residues amongst the methyltransferase domains within this classification of methyltransferases, alpha-like methyltransferase domain [15]. Figure 15 shows the sequence alignment of the methyltransferases within this nidovirales-like methyltransferase family.

The following is found to be conserved within the methyltransferase domain of NSP14 in SARS-CoV: L6079, K6098, Y6099, C6111, P6141, L6155, D6160, D6175, E6204, K6227, F6252, S6259, G6319. The following is found to be conserved within the methyltransferase domain of NSP16 in SARS-CoV: L6802, K6821, Y6822, C6826, P6855, L6869, D6874, D6889, E6922, K6945, F6968, S6975, G7031. The following is found to be conserved within the methyltransferase domain of NSP15 in the Berne virus: L6615, K6633, Y6634, C6638, P6666, L6679, D6684, D6693, E6719, K6737, F6761, S6768, G6824.

When comparing percent identity amongst the sequences, the values observed ranged from 10% to 25%. When NSP14 (SARS-CoV) is compared to NSP16 (SARS-CoV), 12.75% identity is observed. When NSP14 (SARS-CoV) is compared to NSP15 (Berne), 10.57% identity is observed. When NSP16 (SARS-CoV) is compared to NSP15 (Berne), 25.28% identity is observed.

### 4.4. Mononegavirus-like Methyltransferase Domain Family

#### 4.4.1. Protein L (P28887) *Human Respiratory Syncytial Virus A*

**Figure 16:**
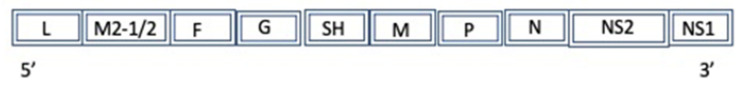
A diagram of the simplified genome for the *Human Respiratory Syncytial virus* A: L (L protein), M2-1/2 (Protein M2-1), F (fusion glycoprotein), G (major surface glycoprotein), SH (small hydrophobic protein), M (matrix protein), P (phosphoprotein), N (nucleocapsid), NS (non-structural protein).

*HRSV* is a negative-sense ssRNA that encodes for a total of 11 proteins. The following are coded: major nucleocapsid protein (N), nucleocapsid phosphoprotein (P), transcription processivity factor M2-1 and 2, L protein, large glycoprotein (G), fusion glycoprotein (F), small hydrophobic protein (SH), non-structural proteins 1 and 2, and non-glycosylated M protein. Figure 16 displays a simplified diagram of the full *HRSV* genome. The L protein assumes the role of viral polymerase to aid in the replication of the viral genome.

There are key motifs present within mononegavirus-like methyltransferase domains that are seen to be pertinent to efficient catalytic activity: GxGxG and KDKE tetrad. The KDKE tetrad can be seen to catalyze the transfer of the methyl group from SAM to its substrate. The glycine-rich domain can be characteristic of the SAM-binding site. This motif usually sits somewhere close to the center of the SAM molecule. The GxGxG can be defined as G1853/E1854/G1855/A1856/G1857. This GxGxG motif can be referred to as Motif I. The following residues have been observed to be essential for methyltransferase activity: K1831, D1936, K1973, and E2004 otherwise known as KDKE tetrad. Motif III is usually a conserved aspartic acid or tryptophan. In the case of the *HRSV-A* virus, there is D1912 which is classified under this motif.

#### 4.4.2. GDP-Polyribonucleotidyltransferase (R4X313) *Nishimuro Ledantevirus*

**Figure 17:**
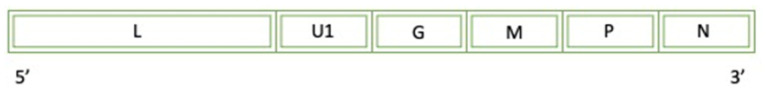
A diagram of the simplified genome for the *Nishimuro ledantevirus* Virus: L (L protein), U1 (U1 protein), G (glycoprotein), M (matrix protein), P (phosphoprotein), (nucleocapsid).

*N. ledantevirus* is a negative-sense single-stranded RNA virus that contains a leader sequence followed by five structural protein genes that are separated by non-transcribed intergenic regions [1]. Figure 17 displays a simplified diagram of the full *Nishimuro Ledantevirus* genome. The five structural proteins are N (nucleoprotein), P (phosphoprotein), M (matrix), G (transmembrane glycoprotein), and L (RNA-dependent RNA polymerase).

Similar to that of the L protein in the *Rabies* virus, this protein is capable of catalyzing the necessary reactions responsible for cap formation of the mRNA. According to domain similarity, this enzyme has the following reported capabilities: GTP phosphohydrolase, PRNTase, RNA-directed RNA polymerase, replicase, transcriptase, guanine-N7-methyltransferase, and nucleoside-2′-O-methyltransferase.

The first step in mRNA capping is a guanosine 5′-triphosphatase (GTPase) that can hydrolyze GTP into GDP and a side product, inorganic phosphate. The GDP acts as an acceptor for the subsequent enzymatic reaction via GDP polyribonucleotidyltransferase (PRNTase). The PRNT domain of the protein recognizes the 5′triphosphate end and forms an intermediate complex between L protein and the pRNA. The pRNA is then transferred to the GDP to form the 5′-terminal cap structure. Since the methyltransferase is non-specific, it can catalyze the formation of cap-0 and cap-1 structures.

Similar to the *HRSV-A* virus, *N. ledantevirus* has the characteristic GxGxG motif and KDKE tetrad to have a functional methyltransferase domain. The GxGxG motif is defined as G1668/D1669/G1670/S1671/G1672. This GxGxG motif can be referred to as Motif I. The KDKE tetrad is defined as K1649/D1760/K1793/E1831. Motif III tends to be a conserved aspartic acid or tryptophan. In the case of the *N. ledantevirus*, there is D1733 which is classified under this motif.

#### 4.4.3. Protein L (P16289) *Rabies*

**Figure 18:**
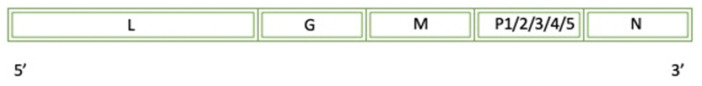
A diagram of the simplified genome for the *Rabies* Virus: L (L protein), G (glycoprotein), M (matrix protein), P (phosphoprotein), N (nucleocapsid).

The *Rabies* virus is a negative-sense single-stranded RNA virus that contains a leader sequence followed by five structural protein genes that are separated by intergenic regions that are not transcribed [19,20]. Figure 18 displays a simplified diagram of the full *Rabies* genome. The five structural proteins are N (nucleoprotein), P (phosphoprotein), M (matrix), G (transmembrane glycoprotein), and L (RNA-dependent RNA polymerase).

Based on the structural similarity to that of the vesicular stomatitis virus, it can be assumed that all enzymatic reactions related to viral RNA synthesis and processing can be linked to the L protein. The RNA-dependent RNA polymerase encoded with the rabies virus will initiate transcription at the 3′end of the *RABV* genome in the order of the following proteins: N, P, M, G, and L. The L protein is heavily involved with cap formation.

The first step in mRNA capping is a guanosine 5′-triphosphatase (GTPase) that can hydrolyze GTP into GDP and a side product, inorganic phosphate. The GDP acts as an acceptor for the subsequent enzymatic reaction via GDP polyribonucleotidyltransferase (PRNTase). The PRNT domain of the L protein recognizes the specific mRNA viral sequence, 5′ppp-AACAG, and forms an intermediate complex between L protein and the pRNA. The pRNA is then transferred to the GDP to form the 5′-terminal cap structure.

The methyltransferase domain is considered to be the motif VI within the L protein sequence. This can be labeled as a mononegavirus-type SAM-dependent methyltransferase. If looking at the full genomic sequence for Protein L, the methyltransferase domain spans residues 1674 to 1871. This methyltransferase can be seen to be utilized for both the N7 of the cap and the 2′OH of the nearby ribose sugar to catalyze the formation of both cap-0 and cap-1 structures.

Similar to the *HRSV-A* virus, the *Rabies* virus has the characteristic GxGxG motif and KDKE tetrad to have a functional methyltransferase domain. The GxGxG motif is defined as G1704/D1705/G1706/S1707/G1708. The KDKE tetrad is defined as K1685/D1796/K1829/E1867. This GxGxG motif can be referred to as Motif I. Motif III is usually a conserved aspartic acid or tryptophan. In the case of the Rabies virus, there is D1770 which is classified under this motif.

#### 4.4.4. Alignment of the Mononegavirales-like Methyltransferase Sequences

**Figure 19:**
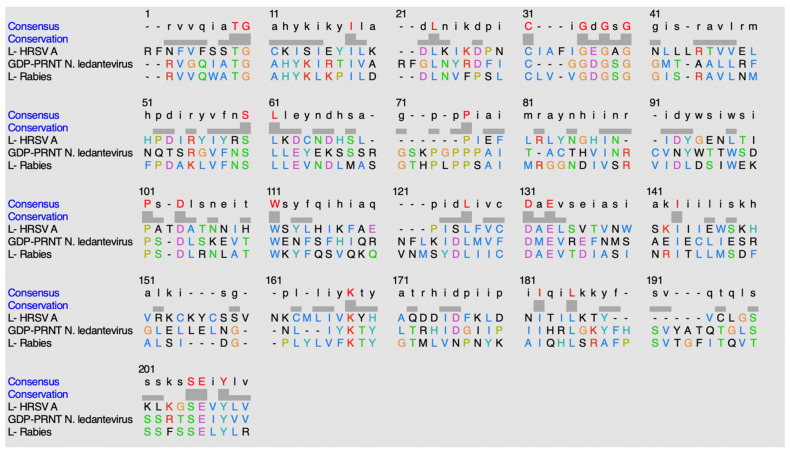
Alignment sequence using ClustalO.

ClustalO was utilized to discover potential conservation of residues amongst the methyltransferase domains within this classification of methyltransferases, mononegavirales-like methyltransferase domain [15]. Figure 19 shows the sequence alignment of the methyltransferases within this mononegavirales-like methyltransferase family.

The following conserved residues are observed in *HRSV-A*: T1828, G1829, I1837, L1841, C1848, G1853, G1855, G1857, S1877, L1878, P1887, P1909, D1912, W1919, L1932, D1936, E1938, I1948, K1973, I1987, L1990, 2003S, E2004, and Y2006. The following conserved residues are observed in *N. ledantevirus*: T1644, G1645, I1653, L1659, C1666, G1668, G1670, G1672, S1691, L1692, P1708, P1731, D1733, W1740, L1756, D1760, E1762, I1772, K1793, I1807, L1810, S1830, E1831, and Y1833. The following conserved residues are observed in the *rabies* virus: T1680, G1681, I1689, L1693, C1700, G1704, G1706, G1708, S1727, L1728, P1744, P1768, D1770, W1776, L1793, D1796, E1798, I1809, K1829, I1843, L1846, S1866, E1867, and Y1869.

When comparing percent identity amongst the sequences, the values observed ranged from 20 to 35%. When GDP-PRNT (*N. ledantevirus*) is compared to Protein L (*rabies*), 34.85% identity is observed. When GDP-PRNT (*N. ledantevirus*) is compared to Protein L (*Human respiratory syncytial virus A*), 20.63% identity is observed. When Protein L (*rabies*) is compared to Protein L (*Human respiratory syncytial virus A*), 23.28% identity is observed.

## 5. Methyltransferases and Their Implication within Disease

As described previously, methyltransferases have the potential to be targeted for drug design. The methyltransferases discussed have had a role in protecting the nascent viral mRNA from host recognition via immune system processes to enable the translation of viral proteins leading to subsequent prolongation of viral life. Inhibition of either cap-0 or cap-1 would lead to the eventual demise of the pathogenic virus.

### 5.1. Inhibiting Cap-0

For those viruses that utilize cap-dependent translation, EIF4E (Eukaryotic initiation factor 4E) is a protein present with the eukaryotic host cell that is responsible for recognizing mRNA and promoting the translation of that strand [21]. More specifically, the EIF4F complex can aid translation in a cap-dependent mechanism. EIF4F is further comprised of the following proteins: EIF4E, EIF4A, and EIF4G [22]. These proteins individually act to do the following: EIF4E binds to the cap, EIF4E acts as a helicase, and EIF4G binds to EIF4E, EIF4A, and PABP (poly-adenylated binding protein). Once the EIF4F complex binds the cap-0 structure, this further recruits the 43S complex and the EIF2-met-trna_i_^Met^-GTP complex [22]. The inhibition of forming this cap structure prevents the translation steps from occurring.

### 5.2. Inhibiting Cap-1

While the Cap-0 structure is methylation of the initial guanine nucleotide at the N7 position, Cap-1 is methylation on the 2′-hydroxyl group of ribose following the cap-0 nucleotide. Without the protection of cap structures, the RNA can be recognized by host processes such as the retinoic acid-inducible gene 1 (RIG-1) which leads to eventual induction of type-1 interferon (IFN). RIG-1 is defined as a cytosolic innate immune receptor that can differentiate cellular self-RNA from that of pathogens. In addition to RIG-1, IFN-induced protein with tetratricopeptide repeats (IFIT-1 and IFIT-5) also can bind exposed 5′-TP on viral mRNA which can induce a similar process [21].

In the situation where a cap-0 has been placed but no 2′O-methylation has occurred (cap-1) then the unmethylated 2′O can be seen as a pathogen-associated molecular pattern (PAMP) where it can be recognized by melanoma differentiation-associated protein 5 (MDA5).

Overall, the binding of this PAMP to a pattern recognition receptor (PRR) can induce a variety of signaling pathways (including IFN-1 production) to rid the cell of the foreign molecule.

As mentioned above, mediators of this pathway include but are not limited to, IFIT, MDA5, and RIG-1 [23,24,25]. Following viral infection, the PAMP is recognized by the PRR of the host system. This binding triggers the signaling pathway to induce IFN-1 formation [21]. Type 1 IFN can bind to the heterodimeric IFN alpha/beta receptor (IFNAR) which is composed of IFNAR1 and 2 subunits. After initial binding, there is further intracellular activity via the JAK-STAT pathway to induce a variety of IFN-stimulated genes. These genes will then transcribe and translate a variety of proteins that act to block stages of viral replication and assist with attenuating viral infection.

RIG-1 and MDA5 are a part of the RIG-1 gene family that participates as PRR to recognize PAMP. IFIT1/5 will be induced by Type 1 IFN induction [21]. The IFIT proteins localize within the cytoplasm and contain tetratricopeptide repeats which is a motif characterized by 34 amino acids. This motif has a helix-turn-helix structure. Generally, proteins with this motif will be involved in some way with the cell cycle. The different IFIT protein types have a different number of TPR motifs which give them a unique function. IFIT1 has been seen to recognize and bind mRNA strands that lack the cap-1 2′O-methylation modification.

For mRNA to begin translation, the cap-binding protein eIF4E is needed. IFIT1 has been seen to compete for binding with CBP eIF4E and has a higher affinity for the cap-0 structure. Additionally, IFIT-1 shows low binding for Cap-1 so if the initial methylation of 2′O occurs, then it is less likely for IFIT-1 to complete its role. Thus, by inhibiting methyltransferase activity, the host cell can activate the RIG-1 system to rid the cell of this pathogenic virus.

### 5.3. Alternative Methods of Escape Related to EIF4E via Viral Mechanisms

In the case of successful cap-inhibition, there are other possible processes in place to be cognizant of when approaching a virus. There are multiple alternative mechanisms to EIF4E that are present: Phosphorylation at S209, 4EBP regulation, alteration of EIF4E expression, EIF4E replacement, EIF4E regulation via binding alternative viral proteins [26].

In adenoviruses, there is a mechanism involving EIF4E dephosphorylation to inhibit cap-dependent translation [26]. Synthesis of protein 100 K allows for binding to EIF4G prevents subsequent binding to EIF4E kinase, MnK1. This prevents EIF4E phosphorylation and halts protein synthesis via this mechanism, but adenoviruses can utilize other means of translation by recruiting a ribosomal complex of 43S and 40S to induce some degree of translation.

In an alternative mechanism, 4EBP is used. 4EBP can also be known as EIF4E-binding protein which acts as translational repressors [19]. In cells infected with the *herpes simplex* virus (*HSV*), *HSV* can form ICP0 protein which promotes the degradation of 4EBP-1 via interaction with a proteasome. If there is less 4EBP-1 available, there is more EIF4E available for translation.

In an alternative mechanism, there is an alteration of EIF4E expression [26]. For example, the *Epstein–Barr* virus can induce EIF4E overexpression via latent membrane protein 1 (LMP1). LMP1 can induce over-expression of EIF4E via C-myc which can lead to the development of nasopharyngeal carcinoma.

In an alternative mechanism, there is a displacement of EIF4E via viral proteins [19]. In the case of *HIV-1*, there is the binding of the DEAD motif to EIF4G and PABP. This induced displacement of EIF4E and allows for efficient translation.

In the final alternative mechanism, viral proteins can be induced to bind EIF4E to alter its function [26]. In the case of *sapovirus*, its viral genome codes for the VPg protein that binds to EIF4E to stimulate its function, thus, increasing viral protein synthesis.

## 6. Potential Methyltransferase Inhibitors That Are Selective for These Methyltransferases

Methyltransferases should be considered as desirable drug targets within antiviral treatments. The methyltransferases discussed play an essential role in forming cap structures needed for the avoidance of the host immune system to prolong the viral life cycle and infection into adjacent cells. By interacting with these methyltransferases and ceasing their activity, one would be able to expose areas of the viral genome that can be recognized by the host immune system, leading to its eventual demise. This section will be divided into experimental inhibitors within positive-sense and negative-sense ssRNA viruses.

### 6.1. Positive-Sense ssRNA MTase Inhibitors

#### 6.1.1. *SARS-CoV* NSP14

**Figure 20:**
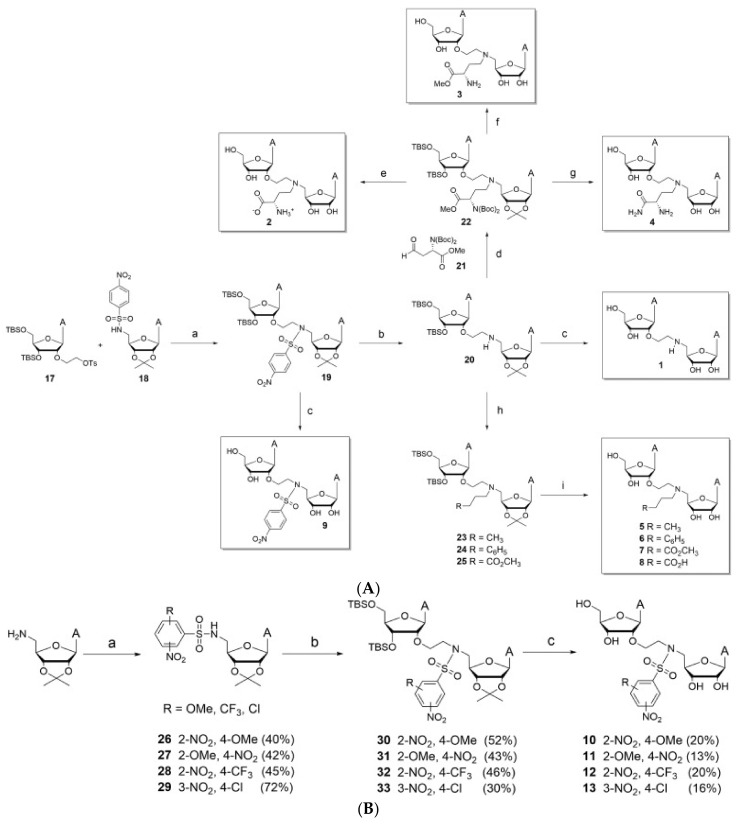

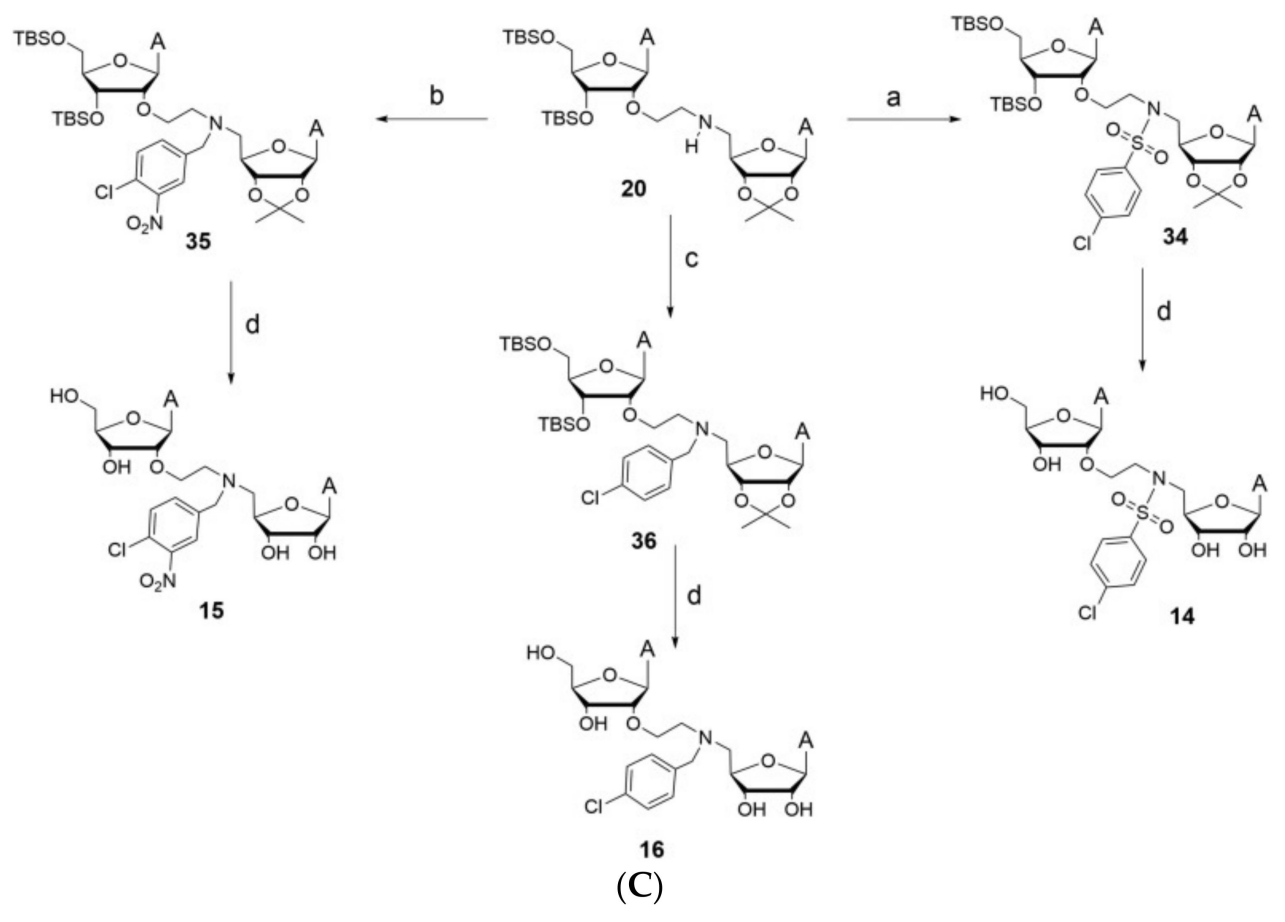
Potential bi-substrate inhibitors that can inhibit both the SAM and mRNA binding pockets. (**A**) This image shows bi-substrate inhibitors that can act on NSP16 of *SARS-CoV-2*. (**B**) This image shows additional bi-substrate inhibitors that can act on NSP16 of *SARS-CoV-2*. (**C**) This image shows bi-substrate inhibitors that can act on NSP16 of *SARS-CoV-2*.

The following substrates are based on the concept of creating a singular molecule with bifunctionality that can bind to both the mRNA binding pocket and SAM binding pocket [27]. The design is meant to mimic that of the transition state of Cap-1 formation on RNA within *SARS-CoV* with an ethyl linker connecting both groups with variations within the N atom substituent contained in the linker. Figure 20a–c provide methods of synthesizing the possible bi-substrate inhibitors. Table 5 and Table 6 show different IC50 values to demonstrate efficacy compared to Sinefungin.

#### 6.1.2. *SARS-CoV* NSP16

**Figure 21:**
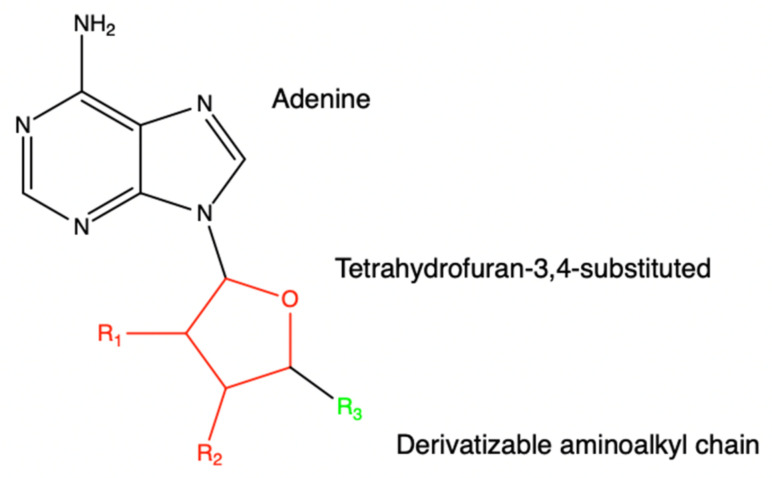
This is a general scaffold design for methyltransferase inhibitors of SARS-CoV NSP16. Various R-groups have been tested through in silico screening and can be found in the paper referenced.

The following molecules are observed to act as inhibitors for the NSP16 belonging to *SARS-CoV* [28]. For NSP16 to utilize its role as a methyltransferase, SAM binds to the SAM-binding pocket to transfer its methyl group. The inspiration for these analogs stems from SAM, SAH, and sinefungin. Figure 21 shows a general scaffold design for NSP16 inhibitors. The binding of SAM, SAH, or sinefungin to its pocket does not affect the adjacent RNA molecule nor its binding pocket so similar analogs with potential inhibitory activity can be utilized to form an effective inhibitor. While no IC50 or EC50 values were recorded for these molecules, other factors were recorded and shown in Table 7 via QikProp and Glide docking scores such as QPlogS, QlogBB, QlogKp, QPlogKhsa, and docking score.

The following compounds are excluded from further testing/results due to the presence of a sulfonium ion that displayed false impressions of potential activity: 144802777, 25245977, and 140671722. This sulfonium ion was labeled as “PAINS” or “Pan-assay interference compounds”. This “PAINS” label refers to molecules that can give false-positive results within screening modalities.

The *Berne* virus has not been as extensively studied as that of the coronaviruses, however, *Berne’s* NSP15 is within the *Toroviridae* family which is under the umbrella of the *Nidovirales* order. Therefore, since *coronaviridae* and *toroviridae* are somewhat similar, it can be proposed that there is potential for cross-reactivity with these inhibitors in *toroviridae* viruses.

#### 6.1.3. *Zika* NS5

**Figure 22:**
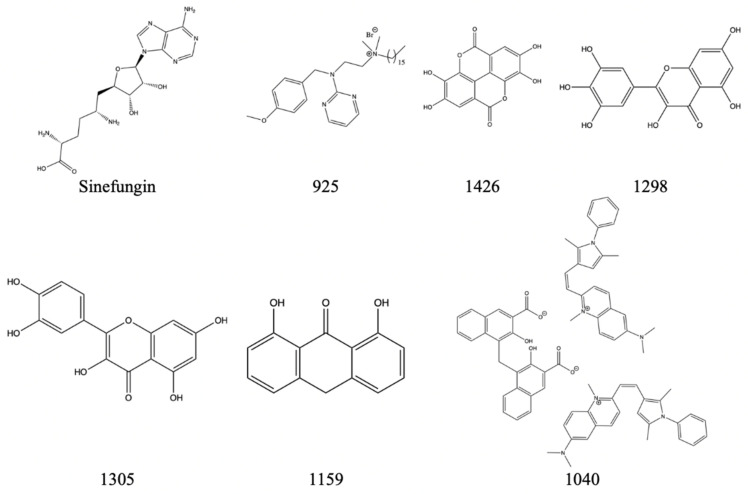
The above inhibitors have the potential to inhibit DENV-3′s NS5 methyltransferase domain and have the potential to inhibit Zika’s NS5.

The following compounds are shown to have some inhibitory activity against the NS5 methyltransferase domain from the *Dengue-3* virus (*DENV-3*) [29,30]. Both the *Zika* virus and *dengue* virus are within the same genus, *flavivirus*, so one can propose that there is a high similarity amongst the NS5 protein and domain. Similar to *Berne* and *SARS-CoV*, as mentioned above, there is potential for cross-reactivity with the *Zika* virus if the drugs were introduced to the virus. Figure 22 shows possible inhibitors for NS5’s methyltransferase along with corresponding IC50 values in Table 8.

#### 6.1.4. Rubella, Semliki, Hepatitis E Genotype 1

**Figure 23:**
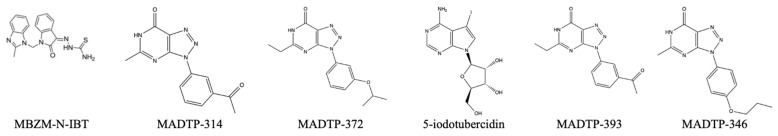
Reported inhibitors of the Semliki NSP1 methyltransferase domain.

This group of viruses contains proteins with very similar methyltransferase domains that can be described to be alphavirus-like methyltransferases based on sequence and structure [31]. While the structures below, in Figure 23, seem to be solely targeted toward NSP1 of the Semliki virus, there might be possible activity amongst the Hepatitis E Genotype 1 and Rubella virus methyltransferase domains. There is currently no EC50 value information available for these drugs.

### 6.2. Negative-Sense ssRNA MTase Inhibitors

**Figure 24:**
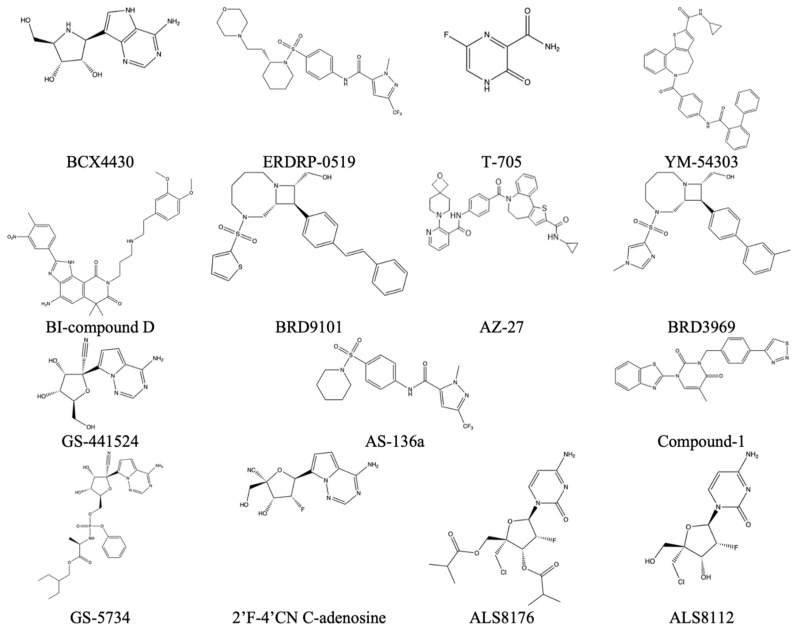
Reported inhibitors of methyltransferases from the mononegavirales order [31].

The following inhibitors, in Figure 24, were seen to have some potential in inhibition of viral methyltransferases within negative-sense ssRNA viruses [32].

#### 6.2.1. Nucleoside Analogs

A subgroup of these inhibitors can be labeled as 2′-fluoronucleoside analogs with inhibition of the *RSV* Polymerase. This group comprises ALS-8112, ALS-8176, and 2′F-4′CN C-adenosine. These work by halting the progression of replication by acting as bases. It can be proposed that halting this domain of the L protein can drastically affect the ability of the methyltransferase domain in the same protein.

#### 6.2.2. Non-Nucleoside Inhibitors

This group of inhibitors is comprised of BI-compound D, YM-53403, AZ-27, Compound-1, BRD9101, BRD3969.

#### 6.2.3. Measles L Protein Inhibitor

This group of inhibitors is labeled as molecules able to inhibit the L protein of the measles virus. While not directly related to that of *HRSV*, there might be cross-reactivity with it due to high similarity amongst sequences and domains. The following inhibitors comprise this group: ERDRP-0519 and AS-136a.

#### 6.2.4. Ebola L Protein Inhibitor

Similar to that of the measles inhibitor section, Ebola has a high sequence similarity and domain structure to that of HRSV [31]. The following inhibitors, found in Table 9, comprise this group: T-705, BCX4430, GS-441524, and GS-5734.

## 7. Conclusions

While the variety of methyltransferase domains is immense, both in structure and specificity for different substrates, there is significant conservation of residues that are needed to maintain optimal functionality. Once a clear understanding of a virus’ methyltransferase is understood, one could develop new inhibitors or variations of existing inhibitors to target them. Vaccination efforts in recent years have exponentially increased, as we have seen with the recent SARS-CoV-2 pandemic. It is important to note that while vaccinations are extremely helpful in teaching the body how to develop an immune response against the spike proteins or proteins involved with entry into the host cell, they may not prove the same efficacy against variants. The outermost proteins of viruses may deviate amongst variations but key residues seem to be conserved when observing the main viral proteins, such as methyltransferases. Understanding these concepts is key to the future of drug design to help us in the case of a future pandemic.

## Figures and Tables

**Table 1:** Summary of the Baltimore Classification System [1].

Class	Genetic Material	Viral Examples
Class 1	dsDNA	*Poxviruses*
Class 2	ssDNA	*Parvoviruses*
Class 3	dsRNA	*Duplornaviricota*
Class 4	(+)-ssRNA	*Kitrinoviricota*
Class 5	(–)-ssRNA	*Orthornavirae*
Class 6	ssRNA-RT	*Revtraviricetes*
Class 7	dsDNA-RT	*Hepadnaviridae*

**Table 2:** Summary of methyltransferase class type and their respective names [6].

Methyltransferase Structural Classes	
Class Category	Name of Methyltransferase Family
Class 1	Rossman-like alpha/beta
Class 2	TIM beta/alpha-barrel alpha/beta
Class 3	Tetrapyrrole methylase alpha/beta
Class 4	SPOUT alpha/beta
Class 5	SET domain all-beta
Class 6	Transmembrane all alpha
Class 7	DNA/RNA-binding 3-helical bundle all alpha
Class 8	SSo0622-like alpha + beta
Class 9	Thymidylate synthetase alpha + beta

**Table 3:** List of methyltransferases and corresponding viral example with Uniprot reference ID [7].

	Characteristics of Single-Strand RNA Virus Methyltransferases		
	Name of Methyltransferase	Viral Example	Uniprot ID
Positive-sense ssRNA	NSP14	*SARS-CoV*	P0C6X7
	NSP16	*SARS-CoV*	P0C6X7
	NSP15	*Berne Virus*	P0C6V7
	NS5	*Zika Virus*	A0A024B7W1
	P150	*Rubella Virus*	Q86500
	NSP1	*Semliki Forest Virus*	P08411
	pORF1	*Hepatitis E Genotype 1*	Q81862
Negative-sense ssRNA	GDP-polyribonucleotidyltransferase	*Nishimuro ledantevirus*	R4X313
	L protein	*Rabies virus*	P16289
	L protein	*Human Respiratory Syncytial Virus A*	P28887

**Table 4:** List of methyltransferases with the general role, function, substrate association, and assigned class [7].

Uniprot ID	Role	Reaction Substrates	Purpose of Reaction	Class
P0C6X7 (NSP14)	This protein has the role of catalyzing the transfer of a methyl group from cofactor SAM to the N7 position of the first guanine nucleotide of mRNA. This methylation forms the cap-0 structure	SAM, lead guanine nucleotide	Using this methyltransferase allows for the formation of Cap-0 structure	Class 1-like SAM-binding methyltransferase superfamily
P0C6X7 (NSP16)	This protein has the role of catalyzing the transfer of a methyl group from cofactor SAM to 2′OH of the ribose following the initial guanine nucleotide of mRNA. This methylation forms the cap-1 structure	SAM, Cap-0	Using this methyltransferase allows for the formation of Cap-1 structure	Class 1-like SAM-binding methyltransferase superfamily
P0C6V7	This is a nidovirus-type SAM-dependent 2′-O-MTase	SAM, free ribose hydroxyl	This reaction forms the Cap-1 structure	Class 1-like SAM-binding methyltransferase superfamily
A0A024B7W1	Catalyzes the methylation of guanine N-7 and ribose 2′-OH	SAM, GTP, cap-0	Formation of Cap-0 and Cap-1 structures	mRNA cap 0–1 NS5-type methyltransferase family
Q86500	This methyltransferase transfers a methyl group from SAM to viral mRNA	SAM, P150/P90 heterodimer, GTP	Formation of cap	Class 1-like SAM-binding methyltransferase superfamily
P08411	Responsible for methylation of GMP at the C7 position	SAM, lead guanine nucleotide	nsP1 is responsible for methylation of the leading guanine nucleotide and then will complex itself to covalently link the cap to the mRNA	Class 1-like SAM-binding methyltransferase superfamily
Q81862	This is an alpha-virus-like methyltransferase	SAM, GTP, GDP	Formation of cap	Class 1-like SAM-binding methyltransferase superfamily
R4X313	This mononegavirus-type SAM-dependent 2′-O-MTase will methylate guanine-N7 and nucleoside-2-OH	SAM, GTP, ribose hydroxyl	Cap formation	Class 1-like SAM-binding methyltransferase superfamily
P16289	This mononegavirus-type SAM-dependent 2′-O-MTase will methylate guanine-N7 and nucleoside-2-OH	SAM, GTP, Ribose-2′-OH	Cap formation	Class 1-like SAM-binding methyltransferase superfamily
P28887	This multifunctional protein has both a ribose 2′-O methylation site and guanine-N7-methylation site. This will form both the Cap-0 and Cap-1 structures. Additionally, the enzyme is SAM-dependent	SAM, cap-0, lead guanine nucleotide	This reaction forms both the cap-0 and cap-1 structures	Class 1-like SAM-binding methyltransferase superfamily

**Table 5:** A table displaying the potential NSP14 inhibitor along with the measured percentage of inhibition of methyltransferase activity when using 50 um.

Name of Molecule	Percentage of Inhibition at 50 um (%)
Sinefungin	98.3 +/− 0.2
1	31.0 +/− 6.8
2	72.0 +/− 1.2
3	30.6 +/− 9.3
4	13.1 +/− 13.3
5	No inhibition detected
6	38.4 +/− 11.7
7	No inhibition detected
8	43.0 +/− 4.0
9	88.6 +/− 1.3
10	96.6 +/− 0.9
11	47.6 +/− 2.8
12	94.6 +/− 1.1
13	97.2 +/− 2.7
14	96.2 +/− 1.5
15	94.0 +/− 1.1
16	75.9 +/− 2.5

**Table 6:** A table displaying the most active NSP14 inhibitors along with the measured IC50 value.

Comparison of IC50 Values with the Most Active Reported Compounds	
Name of molecule	IC50 (um)
Sinefungin	0.36
2	40.6 +/− 3.2
6	55.5 +/− 5.1
9	2.6 +/− 0.2
10	3.9 +/− 0.4
11	70.4 +/− 4.9
12	5.7 +/− 0.5
13	0.6 +/− 0.1
14	1.5 +/− 0.1
15	2.4 +/− 0.2
16	9.9 +/− 0.9

**Table 7:** QPlogS represents predicted aqueous solubility, QlogBB represents predicted log of the brain/blood partition coefficient, QPlogK_p_ represents predicted skin permeability, and QPlogKhsa represents predicted binding to human serum albumin.

Compound (PubChemID)	QPlogS	QlogBB	QPlogKp	QPlogKhsa	Docking Score
44367977	0.20	−1.82	−9.60	−0.99	−12.05
25203154	0.33	−1.56	−9.04	−0.94	−11.83
71008334	0.45	−1.41	−9.96	−0.81	−11.81
14728195	0.45	−1.86	−9.77	−0.92	−11.66
25200440	0.34	−1.96	−9.67	−0.90	−11.63
66856272	0.17	−1.68	−9.27	−0.91	−11.57
44601596	−0.48	−2.52	−8.14	−1.29	−11.50
44601604	−1.32	−2.00	−7.62	−1.16	−11.42
66855668	0.36	−1.54	−9.39	−0.91	−11.04
57126779	−0.96	−2.71	−8.96	−0.94	−11.00
54016655	−1.51	−1.53	−6.63	−0.67	−10.83
57324736	−0.02	−1.92	−9.82	−0.89	−10.11
117805851	−0.87	−1.70	−7.80	−0.78	−10.06
91397803	−0.76	−2.26	−8.43	−0.92	−9.95
71444955	−0.99	−2.60	−7.69	−1.08	−9.94
Sinefungin	−0.39	−3.17	−11.50	−1.29	−8.09

**Table 8:** A table displaying the NS5 inhibitor compound name with its corresponding inhibition percentage at 50 um dose.

Compound Name	Inhibition Percentage at 50 um (%)
Sinefungin	98.3
925	98.9
1426	85
1298	44.9
1305	60.3
1159	37.6
1040	97.8

**Table 9:** Reported virus, corresponding methyltransferase inhibitor, and EC50 value if available. This data is taken from Reference [29].

Virus	Name of Molecule	EC50 (If Applicable) (µm)
HRSV-A	ALS-8112	0.15 µm
	ALS-8176	0.26 µm
	2′F-4′CN C-adenosine	2 µm
	BI-compound D	0.021 µm
	YM-53403	0.72 µm
	AZ-27	0.01 µm
	Compound-1	1.6 µm
	BRD9101	1.7 µm
	BRD3969	1.6 µm
	T-705	260 µm
	BCX4430	11 µm
Measles	AS-136a	0.014 µm
	ERDRP-0519	0.06 µm
Ebola	T-705	67 µm
	BCX4430	11.8 µm
	GS-441524	1.5 µm
	GS-5734	0.07 µm

## Data Availability

Not applicable.
